# Sunlight exposure cannot explain “grue” languages

**DOI:** 10.1038/s41598-023-28280-1

**Published:** 2023-02-01

**Authors:** Joseph L. Hardy, John S. Werner, Terry Regier, Paul Kay, Christina M. Frederick

**Affiliations:** 1 aNUma, Inc, San Rafael, CA 94901 USA; 2 Department of Ophthalmology and Vision Science, University of California, Sacramento, CA 95817 USA; 3 Department of Linguistics, University of California, Berkeley, CA 94720 USA; 4 Cognitive Science Program, University of California, Berkeley, CA 94720 USA; 5 Department of Linguistics, Stanford University, Palo Alto, CA 94305 USA; 6 Sapience Practice, Reno, NV 89502 USA

**Keywords:** Human behaviour, Computational models, Language

**arising from**: M. Josserand et al.; *Scientific Reports* 10.1038/s41598-021-98550-3 (2021).

Recently in this journal, Josserand et al.^1^ argued that high exposure to UV-B light is an important factor in a language’s failing to lexically distinguish green and blue. As noted previously^2,3^, there is a correlation, globally, between (1) societies living in areas with high levels of exposure to UV-B light and (2) the tendency for languages in those societies to lack separate basic color terms for green and blue. Such languages, instead, have either a single term spanning green and blue, referred to as a “grue” term, or a term that merges green and blue with black and other dark shades. It has been proposed that this correlation is caused in part by premature lens aging. While this hypothesis is attractive at first glance, given that UV-B exposure can lead to more rapid yellowing of the eye’s crystalline lens, it overlooks two well-established facts, one about color vision and one about color naming.

First, chromatic adaptation in the visual system compensates for a wide range of prevailing illuminations, ensuring that the perceived color of objects remains relatively constant across different lighting conditions. This phenomenon, known as color constancy^4,5^, is particularly robust at the level of color categorization^6^, which is the level that is important for color naming. Chromatic adaptation and other perceptual mechanisms that support color constancy compensate for changes in the overall retinal illuminance caused by aging of the ocular media of the eye (i.e., “lens brunescence”), as well. The same neural mechanisms that ensure that a green apple appears green in the middle of the day or at sunset ensure that the apple appears green to a young child and an older adult.

Hardy et al.^7^ demonstrated that optical media (crystalline lens, cornea, etc.) brunescence is thoroughly compensated at the level of color naming across the age span of normal, healthy adults. In this study, younger (mean age = 23.2) and older (mean age = 73.9) adults were asked to name a range of standardized color samples. Across the 41-fold range of optical media opacity for participants in this sample, use of the English color term “blue” remained constant (R^2^ = 0.009, *p* = 0.89). These findings indicate that even a very large amount of lens brunescence is insufficient to affect color naming in the manner suggested by Josserand et al.^1^

Second, Berlin and Kay^8^, based on color naming data collected from 20 languages and examination of 78 additional cases from the published literature, concluded that there is a predictable progression through which most languages develop basic color terms. The specific account of color term evolution proposed there has since been significantly modified and simplified, mainly on the basis of color naming data collected in situ from 110 (at the time) unwritten languages^9^. But the initial qualitative finding of Berlin and Kay, namely that there exist substantial predictable regularities across languages in their color naming systems, has been corroborated. For example, no known language has distinct words for blue and green and yet fails to have distinct terms for red and yellow. These cross-language patterns of color naming have been explained in terms that do not rely on lens brunescence but instead on the similarity of colors, communicative need, or a combination of the two^10–13^. Therefore, a more parsimonious explanation for why some languages lack a particular set of color terms is based in need: such a distinction may not be sufficiently important to have arisen in that culture. According to Berlin and Kay^8^, “as a culture becomes technologically more complex, speakers have more frequent need to distinguish objects by their colors” (p. 512). With an increase in the number and variety of trade goods and other objects available in a society, accompanying, for example, the advent of manufacturing, and contact with languages of previously industrialized societies, there is increased pressure on the language used in that society to add color terms. This argument is supported by the observation that distance from the equator (and thus low UV-B exposure), level of technology, and number of basic color terms are positively correlated^14–16^. Thus, the correlation of grue terms with high levels of UV-B exposure could simply reflect the fact that grue terms tend to occur in less technologically complex societies, which tend to be spoken in high UV-B areas. An appeal to lens brunescence is not necessary to explain the geographical distribution of languages with grue terms.

One final consideration concerns historical language change. For example, Old Japanese had a grue term, *ao(i)*, probably appearing between 500 and 650 CE, which now serves as the common term for blue. The present common term for green, *midori*, entered late Old Japanese or Early Middle Japanese sometime after 750^17,18^. Thus, Japanese has shifted from having a grue term to now having separate terms for green and blue. Similarly, but much more recently, the Nafaanra language, spoken in Ghana, had a grue/dark term in 1978 but now has distinct terms for green and blue^19^. We are not aware of any evidence that the amount of UV-B exposure in Japan or Ghana has decreased markedly over the relevant time periods. A more parsimonious explanation is that these two languages have followed a natural pattern of language evolution from fewer to more basic color terms, as the cultural need for a finer-grained color vocabulary increased.

For these reasons, we conclude that normal lens brunescence is unlikely to affect color perception enough to alter color naming and is, in any event, unnecessary to explain patterns of color naming and their geographic distribution. A limitation of the current literature is that little is known about the epidemiology of lens brunescence in high UV-B environments. Further empirical research into the rate of brunescence in geographic areas in question would aid our understanding of this issue.
